# Molecular profiling of basal cell carcinomas in young patients

**DOI:** 10.1186/s12920-021-01030-w

**Published:** 2021-07-20

**Authors:** Marc Abi Karam, Hampig Raphael Kourie, Nadine Jalkh, Cybel Mehawej, Carole Kesrouani, Fady Gh Haddad, Iman Feghaly, Eliane Chouery, Roland Tomb

**Affiliations:** 1 Faculty of Medicine, Saint Joseph University, Beirut, Lebanon; 2 Medical Genetics Unit, Faculty of Medicine, Saint Joseph University, Beirut, Lebanon; 3 Hematology-Oncology Department, Faculty of Medicine, Saint Joseph University, Beirut, Lebanon; 4 Pathology Department, Faculty of Medicine, Saint Joseph University, Beirut, Lebanon; 5 Dermatology Department, Faculty of Medicine, Saint Joseph University, Beirut, Lebanon

**Keywords:** Basal cell carcinoma, NMSC, *PTCH1*, *TP53*, NGS

## Abstract

**Background:**

Basal cell carcinoma (BCC) represents by far the most common non-melanoma skin cancer (NMSC) in the world with an increasing incidence of 3% to 10% per year, especially in patients under the age of 40. While variants in the sonic Hedgehog and cell cycle regulation pathways account for the majority of BCC cases in adults, the molecular etiology of BCC in young patients is unelucidated yet. This study aims to investigate the molecular profile of BCC in the young population.

**Methods:**

28 tumors belonging to 25 Lebanese patients under the age of 40, presenting different stages of BCC and diagnosed at Hôtel Dieu de France—Saint Joseph University Medical Center were included in this study. A selected panel of 150 genes involved in cancer was analyzed by Next Generation Sequencing (NGS) in the 28 included tumors.

**Results:**

Genetic variants detected in more than 5% of the reads, with a sequencing depth ≥ 50x, were selected. Two hundred and two genetic variants in 48 different genes were detected, with an overall average sequencing depth of 1069x. Among the 28 studied tumors, 18 (64.3%) show variations in the *PTCH1* gene, 6 (21.4%) in *TP53* and 3 (10.7%) in *SMO*.

**Conclusions:**

This is the first study reporting NGS-based analysis of BCC in a cohort of young patients. Our results highlight the involvement of the hedgehog and cell cycle regulation pathways in the genesis of BCC in the general population. The inclusion of a larger cohort of young patients is needed to confirm our findings.

**Supplementary Information:**

The online version contains supplementary material available at 10.1186/s12920-021-01030-w.

## Background

Skin cancers are a heterogeneous group of cancers including Melanoma Skin Cancer (MSC) and Non Melanoma Skin Cancer (NMSC). NMSCs are 20 times more common than MSCs and their incidence is still increasing especially in northern Australia due to increased sun exposure [1]. With 1.6 million new cases of NMSC reported in 2012 by the American Cancer Society [2] and an incidence that is expected to double in the next 30 years [3], this type of skin cancers remains a public health issue worldwide. Basal cell carcinoma (BCC), a subtype of NMSC, is a common skin cancer arising from the basal layer of epidermis and its appendages.

Despite the fact that NMSC is excluded from cancer-registry statistics and that the absolute incidence of BCC is difficult to determine, the highest NMSC rates are found in the elderly population, with an incidence reaching approximatively 2000 cases per 100000 inhabitants. This could be due to the accumulation throughout life of somatic variants and to potential damages caused by chronic sun exposure. Many epidemiologic studies showed an increasing incidence of BCC in the general population [4, 5]. That said, a higher incidence of this disease was also reported, in 2014, in young women [6, 7]. The American Cancer Society however estimates that in 2012, 5.4 million cases of NMSCs were diagnosed in 3.3 million people, of which approximately 8 in 10 cases would have been BCC [8].

Additional risk factors for BCC in the general population include HPV (Human Papilloma Virus) infection, *xeroderma pigmentosum*, albinism, chemical carcinogens (arsenic and coal tar), and ionizing radiation. Furthermore, people with specific physical characteristics such as blond or red hair, blue or green eyes, and light skin color are more prone to skin cancer [9, 10].

BCC results, in most cases, from an hyperactivation of the Hedgehog (Hh) signaling cascade. *PTCH1* inactivation/inhibition accounts for ~ 70% of BCCs while *SMO* activation for ~ 20% [11, 12]. In addition to the genes involved in Hh, cell cycle regulating genes such as *TP53* and *MYCN* also contribute to BCC pathogenesis [13].

The majority of BCCs are sporadic. To date, four studies investigated the molecular bases of BCC in small or large cohorts, all belonging to the elderly population. Different approaches including Sanger sequencing of selected genes or Whole Exome Sequencing (WES) were used. Somatic variants in *PTCH1, SMO, SUFU, TP53* and *MYCN* genes were found to be involved in the pathogenesis of sporadic BCC in the elderly population, with *PTCH1* and *TP53* being the two most commonly mutated genes in these patients [13–16].

In rare cases, germline variants in the *PTCH1*, *PTCH2*, *SUFU* genes may be responsible for an inherited form of BCC known as Gorlin Syndrome (MIM # 109400) [17]. The contribution of the *BAP1* gene to some hereditary forms of BCC has also been demonstrated [18].

Up to now, all reported studies included mainly patients belonging to the elderly population which is the most commonly affected with BCC. Molecular profile characteristics of BCC in young patients are yet to be identified. The current study evaluates the molecular basis of BCCs in young Lebanese patients and compares the obtained results to published data.

## Methods

### Patients selection and characteristics

This is a 10-year retrospective study undertaken at the *Hôtel Dieu de France* (*HDF*) University Hospital of Saint Joseph University, Beirut—Lebanon. Approval to conduct this research was obtained from the Ethics Committee of Saint Joseph University, Beirut—Lebanon. The medical records of 62 people under the age of 40, with different BCC stages and having undergone tumor tissue resection were found in the hospital’s database. However, following a personal contact for all patients, we were able to collect epidemiological and clinical data from only 40 patients.

**Fig. 1:**
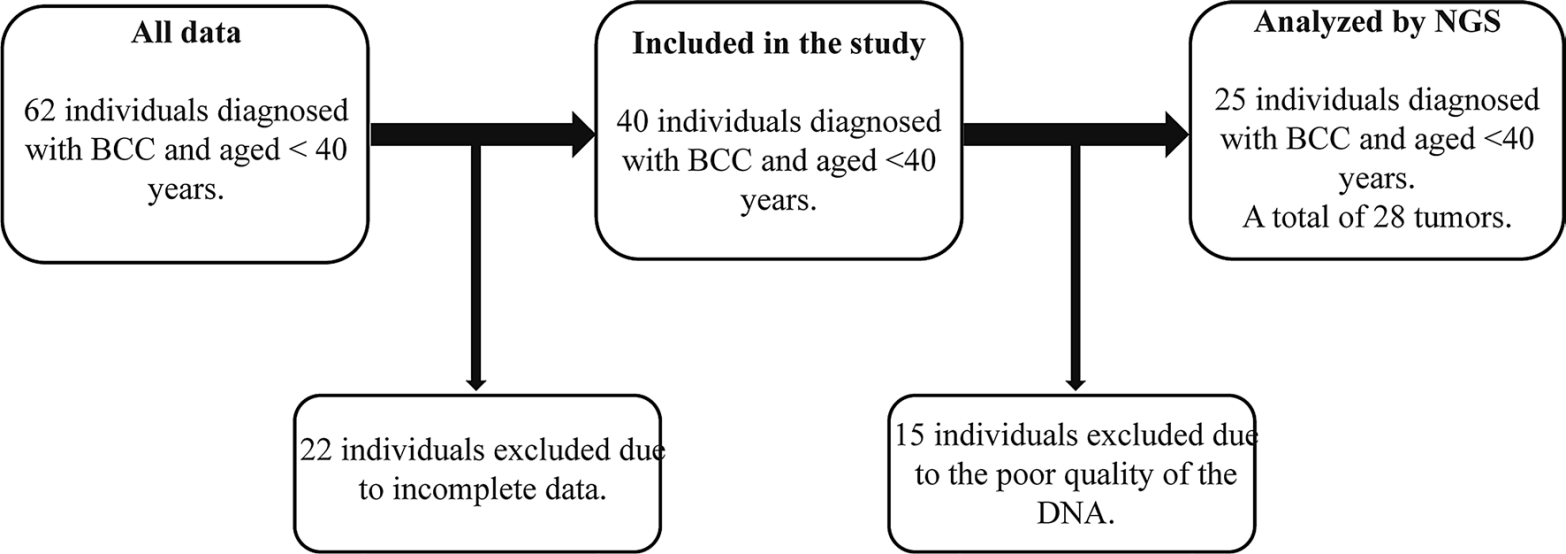
Approach to the selection of patients included in this study

The resected tumor biopsies fixed in 10% formalin, and preserved in paraffin blocks, were re-evaluated at the anatomopathological department of HDF. The tumor cellularity was evaluated after analysis of the slides stained with hematoxylin and eosin. From each paraffin block, 10 sections of 5 μm thickness were prepared and put in Eppendorf tubes at room temperature. After DNA extraction, the DNA quality of 18 biopsies turned out to be poor. Again, verification of tumor cellularity was carried out for these 18 cases and new sections were prepared. A total of 28 tumor tissues belonging to 25 patients were finally included to be analyzed by NGS techniques (Fig. 1). The three extra tumors are independent BCC tumors from three of these patients resected at different intervals of time (1–3 years).

### DNA extraction and quality assessment

DNA was extracted from FFPE samples using the QIAmp DNA FFPE Tissue Kit (Qiagen, Hilden, Germany) according to the manufacturer's instructions. Quality and quantity of DNA were assessed using Nano-Drop ND-1000 Spectrophotometer (Nano Drop Technologies, Wilmington, DE), agarose gel electrophoresis and quantitative polymerase chain reactions (qPCR).

### Genes of interest

Based on a deep literature review, 25 candidate genes are known to be involved in the genesis of BCC in the elderly population, these are the following genes: *TP53*, *NRAS*, *KRAS*, *HRAS*, *BRAF*, *CTNNB1*, *PTCH1*, *SMO*, *SUFUH*, *GLI1*, *PIK3CA*, *RAC1*, *FBXW7*, *RB1*, *CDKN2A*, *ARID1A*, *NOTCH1*, *CASP8*, *NOTCH2*, *MYCN*, *STK19*, *LATS1*, *ERBB2*, *PPP6C* and *PTPN14.*

For our study, we selected the "Solid Tumor Panel", available from Centogene AG (Rostock, Germany), which is designed for the study of somatic variants in solid tumors and contains 150 genes including 19 of the genes listed above, thus allowing the identification of novel candidates involved in BCC in the young population (Additional file 1: Appendix 1).

### NGS and bioinformatics analysis

Briefly, genomic DNA is enzymatically fragmented and DNA capture with probes targeting the coding regions of the panel genes and known relevant hotspot regions for solid tumors is performed (gene list is described in Additional file 1: Appendix 1). The libraries are subsequently sequenced on a MiSeq Illumina platform to achieve at least 200 × depth of coverage for 97% of the targeted region. Raw sequence data analysis, including base calling, demultiplexing, alignment to the hg19 human reference genome (Genome Reference Consortium GRCh37) and variant calling (single nucleotide variants and InDels) was performed using an *in-house* pipeline. Briefly, sequencing reads were aligned to the hg19/b37 reference genome using the Burrows-Wheeler Aligner (BWA) package v0.6.1 [19]. Local realignment of the mapped reads around potential insertion/deletion (Indel) sites was carried out with the Genome Analysis Tool Kit (GATK) v1.6 [20]. Duplicate reads were marked using Picard v1.62. Additional BAM file manipulations were performed with Samtools 0.1.18 [21]. Base quality (Phred scale) scores were recalibrated using GATK’s covariance recalibration. SNP and Indel variants called using the GATK Unified Genotyper for each sample [22]. Variants were called using high stringency settings and annotated with VarAFT software 1.61 [23] containing information from dbSNP147 and ExAC (http://exac.broadinstitute.org/). Relevant genetic variants were only selected if their depth is ≥ 50 × with a percentage of reads ≥ 5% (sequencing quality assessment). All identified variants were evaluated with respect to their pathogenicity and causality, and categorized into four classes (Tier I, variants with strong clinical significance; Tier II, variants with potential clinical significance; Tier III, variants of unknown clinical significance; and Tier IV, variants deemed benign or likely benign) based on the guidelines of the Association for Molecular Pathology (AMP), American Society of Clinical Oncology (ASCO) and College of American Pathologists (CAP) [24]. In order to select the deleterious variants, variants with a frequency greater than 0.1% in public databases (including gnomAD and 1000 Genomes) were filtered out in order to only select rare variants that might be relevant to BCC [25]. Furthermore, due to the challenging interpretation of variants found in non-coding areas such as UTRs and intronic regions, these were discarded from our analysis unless their implication in the disease was previously reported.

The identified genetic variations were statistically studied by a simple descriptive analysis (frequency, percentages) and compared to the genetic variations data in the literature. Association studies between patient's age, sex, skin color, sun exposure, tumor location, histology, family history of cancer and the identified variants were evaluated by χ2 and Mann–Whitney tests.

## Results

### Population demographics

The studied population included 25 individuals (P1-P25) with any BCC stage and having undergone a tumor tissue resection. All patients were under the age of 40 with a mean age at which the tumor was resected for biopsy equals 33.5 years (range 24–40). Among these patients, 56% are men and 44% are women. Two risk factors, exposure to sun and the skin color, known to be involved in BCC development have been evaluated (Table 1).

**Table 1:** Characteristics of the 25 BCC included patients

Characteristics	Results
Mean age at which the tumor was resected for biopsy	33.5 years
Sex	14 (56%) men 11 (44%) women
Number of patients with an occupation exposing to the sun	9 (36%)
Number of patients with fair skin color	13 (52%)
Number of patients with dark skin color	12 (48%)
Number of patients who tan frequently	13 (52%)
Number of patients with a family history of skin cancer	4 (16%)
Number of patients with other types of cancer in the family	10 (40%)

Sixteen percent of the studied population have in the same family a history of skin cancer (patients P5, P14, P20 and P22) and 40% present other types of cancer including: 4 cases of breast cancer (mothers of P4, P13, P17 and P18), 3 cases of colon cancer (P1, P11 and P20), 1 case of liver cancer (P8), 1 case of bladder cancer (P10) and another case of stomach cancer (P19). A total of 13 of the 25 (52%) patients have a family history of cancer including skin or other types of cancer (P20 with a history of skin and colon cancer) (Additional file 2: Appendix 2).

### Characteristics of patients’ BCC lesions

Tumors were distributed over different parts of the body. They were observed on the head and neck (61%), breast (14%), thorax (14%) and on the scalp (7%). Among the tumors present on the head and neck, 41% are located on the cheeks, 24% on the ears, 18% on the nose, 12% on the upper lips and 6% on the forehead.

Tumors were divided into 5 types: nodular (27%), infiltrative (19%), pigmented (12%), superficial (23%) and indeterminate (19%) (Additional file 2: Appendix 2).

### NGS analysis

#### Total variants detected

The global analysis of the generated data showed a 99.9% coverage of the sequenced genes at a minimum sequencing depth of 200 × and an overall average depth of 1069x. After applying the filtering strategy detailed above, 202 variants were detected in 48 genes in all 25 patients (Table 2) with an average of 3.6 variations per patient (range 2—23). Of these variants, 8.9% are classified as Tier I and 91% as Tier II; they include 186 loss of function (splicing, frameshift or nonsense) and 16 missense variants (Additional file 2: Appendix 2).

**Table 2:** The list of all variants found and their recurrence in the mutated genes in our cohort

	Genes	Number of patients with variants (percentages)
Genes of interest	*PTCH1*	17 (64.3%)
	*TP53*	6 (21.4%)
	*SMO*	3 (10.7%)
	*RB1*	2 (7.1%)
	*MYCN*	1 (3.6%)
Additional genes	*RBM10*	23 (82.1%)
	*PALB2*	16 (57.1%)
	*ATRX*	15 (53.6%)
	*APC*	11 (39.3%)
	*TSC1*	8 (28%)
	*KMT2C*	7 (25%)
	*ATM*	6 (21.5%)
	*FANCC, MSH6, NBN*	4 (14.3%)
	*BRCA2*, *CREBBP*, *EZH2*, *KMT2D*, *NF1*	3 (10.7%)
	*BMPR1A*, *BRCA1*, *RBB4*, *RAD50*	2 (7.1%)
	*AR*, *ASXL1*, *ATR*, *AXIN2*, *CDH1*, *EP300*, *ERCC2*, *FANCA*, *FGFR1*, *IRF1*, *JAK3*, *KDM5C*, *KMT2A, MLH1*, *MUTYH*, *PMS2*, *PPP2R1A*, *PTPN11*, *RAD51B*, *RBBP8*, *RHOA*, *SEDT2*, *SMARCB1*, *XRCC2*	1 (3.6%)

#### Variants potentially involved in BCC pathogenesis

In order to select variants that could have directly contributed to the development and progression of BCC, we thoroughly analyzed the genes included in the “Somatic tumor genes panel” (Additional file 1: Appendix 1) and selected for each patient, the variant with the highest percentage of sequencing reads. Out of 25 patients, 19 carried variants in the analyzed genes: 16 in the *PTCH1* gene and 3 (P5, P7 and P14) in *ASXL1*, *SMO* and *TP53* genes, respectively. The selected variants for each sample are presented in Additional file 2: Appendix 2. Briefly, *PTCH1* variants co-occurred with an additional variant in *TP53* in 4 patients (P6, P9, P13 and P19) or with a variant in *SMO* gene in one patient (P15). Furthermore, 2 patients (P1 and P18) have each two different *PTCH1* variants (Table 3).

**Table 3:** List of variants likely to be the driver variants

Patient	Tumor	Gene	Mutation	% of reads	Classification	gnomAD
P1	7111 12	*PTCH1*	c.3261dup	11.6% of 1537	Tier 1	–
			p.(Ala1088Argfs*57)			
		*PTCH1*	c.1347 + 1G > A	14.1% of 978	Tier 1	–
P2	10539 13	*PTCH1*	c.413_429dup	25.8% of 538	Tier 1	–
			p.(Arg144Valfs*3)			
P3	656 09	*PTCH1*	c.3499_3500delinsAA	30.4% of 1281	Tier 1	–
			p.(Gly1167Lys)			
P4	9219 17	*PTCH1*	c.310dupG	51.3% of 3118	Tier 1	–
			p.(Val104Glufs*36)			
	8608 17	*PTCH1*	c.310dupG	63.8% of 2933	Tier 1	–
			p.(Val104Glufs*36)			
P5	3496 15	*ASXL1*	c.2893C > T	18.7% of 2578	Tier 2	1.19E-05
			p.(Arg965*)			
P6	6028 04	*PTCH1*	c.1011G > A	44.3% of 1509	Tier 1	–
			p.(Trp337*)			
		*TP53*	c.535C > T	27.9% of 1665	Tier 2	–
			p.(His179Tyr)			
P7	7493 07	*SMO*	c.1604G > T	19.5% of 1592	Tier 2	–
			p.(Trp535Leu)			
P9	1833 07	*PTCH1*	c.3499G > A	15.5% of 1033	Tier 1	–
			p.(Gly1167Arg)			
		*TP53*	c.524G > A	14.1% of 997	Tier 2	3.98E-06
			p.(Arg175His)			
P10	1124 15	*PTCH1*	c.2917C > T	35% of 940	Tier 1	–
			p.(Gln973*)			
P11	3480 15	*PTCH1*	c.1223_1225delinsTTT p.(His408_Gln409delinsLeu*)	13.7% of 831	Tier 1	–
P13	4707 07	*PTCH1*	c.2560_2560 + 1delinsAA	17.5% of 1993	Tier 1	–
		*TP53*	c.948_949delinsTT	17.2% of 3057	Tier 2	–
			p.(Gln317*)			
P14	8579 10	*TP53*	c.1024C > T	18.3% of 1561	Tier 2	–
			p.(Arg342*)			
P15	12702 15	*SMO*	c.1604G > T	26.2% of 1231	Tier 2	–
			p.(Trp535Leu)			
	15012 15	*PTCH1*	c.394_394 + 1delinsAA	28.8% of 1147	Tier 1	–
P18	11975 16	*PTCH1*	c.2208del	11.4% of 1675	Tier 1	–
			p.(Glu737Argfs*9)			
	13596 14	*PTCH1*	c.2557C > T	50.6% of 806	Tier 1	–
			p.(Gln853*)			
P19	6062 09	*PTCH1*	c.2250 + 1G > A	35.3% of 920	Tier 1	–
		*TP53*	c.920-1G > A	20.2% of 1635	Tier 2	–
		*TP53*	c.470_471dupTC	25.4% of 1382	Tier 2	–
			p.(Arg158Serfs*13)			
P20	12537 08	*PTCH1*	c.4180C > T	27.2% of 1913	Tier 2	3.19E-05
			p.(Arg1394*)			
P22	13254 16	*PTCH1*	c.1347 + 2T > A	61.8% of 1650	Tier 1	–
P24	3418 12	*PTCH1*	c.394 + 1G > A	23.7% of 3217	Tier 1	–
P25	10999 08	*PTCH1*	c.1348-1G > T	5.3% of 1391	Tier 1	–

Tier I, variants with strong clinical significance; Tier II, variants with potential clinical significance; –, Not found in gnomAD, numbers in gnomAD represent the frequencies of each variant if found in this database

Our data also showed that 2 patients (P1 and P22) have each one variant at the same splicing site in the *PTCH1* gene and that two other patients (P7 and P15) have the same variant in the *SMO* gene (Table 3).

Six remaining patients (P8, P12, P16, P17, P21 and P23) did not have any variants in the Hh signaling pathway nor in the other studied genes and remained genetically uncharacterized.

For three patients (P4, P15 and P18), two different tumors (from different locations) per patient were sequenced and compared (Table 3 and Additional file 2: Appendix 2). Patient P4 presents, in both tumors, the same variant in *PTCH1* gene, detected at a high frequency of reads (51.3% and 63.8%), thus suggesting that it could potentially be a germline variant involved in Gorlin syndrome associated with BCC in patient P4. However, this hypothesis was unfortunately not confirmed due to inaccessibility of further samples from this patient and to his unavailability for a more thorough clinical evaluation. On the other hand, patient P15 presents in each tumor different variants, of which a variant in *PTCH1* and another in *SMO* are considered as responsible for BCC in the first and second tumors, respectively. Patient P18 presents in each tumor a different selected variant but both in *PTCH1* and both are considered as involved in BCC*.* In addition, the molecular profiles comparison of the two tumors, for each patient, showed genetic heterogeneity, thus highlighting the complexity of genomic data interpretation in tumoral samples.

On the other hand, the evaluation of genes involved in the *RAS* signaling pathway (including the *NRAS*, *HRAS* and *KRAS* genes), a pathway involved in several cancer types, did not reveal any variant in our cohort.

#### Association studies

No correlation was observed between the number of BCC lesions or their locations and the number of variants in each individual. This lack of correlation was verified by the Wilcoxon Mann–Whitney statistical study which showed a *p value* of 0.495 and a variants median for each BCC lesion equal to 5. Additionally, no correlation was found between the characteristics (skin color, tanning, sun exposure, family history of cancers) of the individuals and the mutated genes.

In parallel, variants identified did not seem to cluster at specific sites in the genes of interest. However, this size of our cohort does not enable enough statistical power to judge.

## Discussion

This is the first NGS-based study targeting BCC patients under the age of 40 and aiming to evaluate the molecular basis of this disease in the young population. NGS data was analyzed for the identification of variants that could potentially be involved in BCC genesis and transformation, in the 25 young patients included in this study (28 tumors). A comparison between the molecular profile of the young patients herein studied and previously reported patients was also performed.

Among the 25 studied patients, candidate variants were identified in 19 affected individuals: 16 in *PTCH1* gene and the 3 remaining each have a variant in *ASXL1*, *SMO* and *TP53* respectively. Among the 16 variants in *PTCH1* gene, 8 are predicted to be loss-of-function variants while the variant p.(Trp535Leu) in *SMO* detected in P15, is an activator of the corresponding protein. It is also known to be a somatic BCC driver [26]. An inhibition of the expression of *PTCH1* or an aberrant activation of *SMO* are known to be factors leading to the development of BCC. As a matter of fact, driver variants in *SMO* and *PTCH1* are considered to be predictive biomarkers for the response of BCC patients to the treatment with Vismodegib [27] (Hh pathway inhibitor). Our study confirms the importance of the molecular diagnosis of BCC patients in guiding their treatment.

In patient P5, a known somatic BCC driver in *SMO* (p.Leu412Phe) [26] was detected but at a very low allele frequency (8.3%). On the other hand, the *ASXL1* gene is mutated in the same patient with the highest percentage of reads. *ASXL1* encodes a protein that interacts with BAP1 to form a functional protein complex [18]. Loss of expression of *BAP1* due to germline variants in this gene has been associated with the development of BCC, uveal and cutaneous melanomas [28]. Further investigations are needed in order to assess the pathogenicity of the detected *ASXL1* variant and its possible involvement in the modulation of BCC severity in this patient.

Of the 25 patients included in this study, 6 patients remain genetically undiagnosed. This could be explained by the presence of variants or loss of alleles (LOH, or Loss of Heterozygosity) in genes not covered by the used technique or in regulatory regions that are not included in our analysis. Indeed, a LOH at chromosome 9q22 (locus containing *PTCH1*) was previously shown to be responsible for sporadic cases of BCC [13]. In addition, certain genes known to be involved in BCC, such as *BAP1*, *PTCH2*, and *SUFU* are not included in the panel of genes chosen for this study. Therefore, genetic evaluation of these genes and searching for LOH at chromosome 9q22 must be carried out in the 6 remaining patients.

Interestingly, 8 samples have the same splicing variant, c.2041 + 1G > C in *TSC1* gene with relatively high read percentages. In a study published in 2002, Wienecke R et al. have shown that the loss or the downregulation of tuberin due to variants in *TSC1* and *TSC2* genes could contribute to tumor proliferation [29]. More work is needed to understand the mechanism leading to the occurrence of this variant as well as its role. In addition, *PALB2* an essential gene in repairing homologous recombination is mutated in 16 samples but with a low read percentages. Similarly, 22 patients have the same variant p.(Gln350_Ser356delinsArgAlaLeu*) in *RBM10* gene but with low read percentages. *PALB2* and *RBM10* are involved in DNA repair; their involvement in the tumor process is not surprising. Interestingly, these genes seem to be more frequently mutated in our young cohort compared to others (11% vs ^~^ 32% for *TSC1*, 11% vs ^~^64% for *PALB2*, 7% vs ^~^88% for *RBM10*), as per cbioportal (https://www.cbioportal.org/). However, owing to the small sample size, our findings are not conclusive and need further validation. Moreover, these recurrent variants must be confirmed by other techniques as their presence in low percentages in the majority of the samples might be due to a sequencing artefact that generated false positive variants. Sequencing artefacts, especially C > T/G > A transitions were found to be present with 1–10% allele frequency range in formalin-fixed samples, due to DNA damage [30]. The degree of fragmentation and sequencing artefacts depends on the biopsy age, since long-term storage of formalin-fixed blocks can induce fragmentation due to exposure to environmental conditions [31–33]. Therefore, the high number of transitions with low allele frequency observed in samples P7, P9 and P5 may be attributed to DNA damage.

Four patients (P6, P9, P13 and P19) present, each in the same biopsy, two variants one in *PTCH1* and another in *TP53*. The co-occurrence of variants in these two genes was previously reported in other cases with BCC [15]. Interestingly, reanalysis of the pathology data from these patients showed that patient P6 had an irregular pigmented and nodular BCC that looked like malignant melanoma and was clinically confusing and patient P13 had melanoma. The presence of a collision tumor of malignant melanoma and BCC in a young patient was only reported in one patient but was not genetically studied [34]. Further investigations are needed in order to explore the molecular basis of these collision tumors in young patients.

We were able to include in our molecular study two different tumors from three patients (P4, P15 and P18) presenting different BCC lesions. The comparison of the genetic profiles of the two tumors for the same patient showed the following: a variant in *PTCH1* that could be germinal was detected in P4, thus explaining the occurrence of several BCCs in this patient who was not routinely exposed to the sun. However, the confirmation of the presence of this variant in a nontumoral sample from this patient as well as his clinical reassessment are crucial for the validation of this hypothesis. A genetic heterogeneity was noted in the two biopsies belonging to patients P15 and P18; different variants were identified in *PTCH1* and / or *SMO* genes. We speculate that these patients may carry germline variants in the DNA repair genes rendering them more susceptible to develop recurrent tumors. Indeed, Cho H. et al*.* have shown that germline variants in DNA repair genes—such as *APC*, *BARD1*, *BRCA1*, *BRCA2*, *CDH1*, *CHEK2*, *MLH1*, *MSH2*, *MSH6*, *MUTYH*, *NBN* and *PALB2*- are implicated in recurrent BCCs [35]. Another possibility is that the different variants in *PTCH1* and / or *SMO* may have occurred accidentally and contributed independently to BCC pathogenesis.

Among the patients recruited in this study, 38% present with a family history of cancer (including skin cancer or other). Owing to the fact that the current study focused on the evaluation of variants in tumoral tissues, further investigations are needed in order to assess the possibility of the presence of germline variants in genes involved in cancer in these patients. This is important to enable an accurate genetic counseling and clinical management of individuals carrying variants increasing their susceptibility to develop cancer in these families.

Altogether, comparable to previously reported data in BCC patients older than 40, *PTCH1* was found to be the most frequently mutated gene in the young BCC patients included in this study. Indeed, variants in the *PTCH1* gene were detected in 64.3% of the analyzed tumors; a slightly lower frequency compared to the literature (Table 4) where percentages exceeding 70% have been reported. However, the occurrence of *TP53* variants in our cohort (21.4%) is significantly lower compared to other studies: 61% [13] and 66% [15]; and likewise for *SMO* and *MYCN* variants, as follows: 11% vs 20% and 4.0 vs 30% [13], respectively. The decrease in the frequency of variants herein detected compared to frequencies reported by Jayaraman et al. [15] and Bonilla et al. [13] was unexpected, especially that the technology herein adopted (genetic panel) is characterized by a higher sequencing coverage and reading depth than the WES that was carried out in previous studies. On the other hand, variants in the tumor suppressor gene *RB1* were found in two patients from our cohort. This confirms once more the genetic heterogeneity of skin cancers and highlights the importance of studying larger cohorts of young patients with BCC for a better delineation of the molecular bases of this disease.

**Table 4:** Percentages of the mutated genes in patients with BCC from different studies

Different studies	*PTCH1*	*TP53*	*SMO*	*RB1*	*MYCN*
Reifenberger et al. [14]	50–85%				
Jayaraman et al. [15]	75%	66%	–	–	–
Bonilla et al. [13]	73%	61%	20%	< 1%	30%
Maturo et al. [16]	59%	31%			
Our study	64%	21%	11%	7%	4%

Conclusions: In summary, this is the first study investigating BCC pathogenesis in the young population and reporting the molecular profile of BCC in Lebanese patients.

Our initial findings endorse the involvement of the Hh and cell cycle regulation pathways in the genesis of BCC in the general population and show that this contribution is independent of the age of onset of the disease.

However, complementary analyses such as the validation of identified genetic variants by other techniques or sequencing the non-investigated genomic regions are required depending on each case. Last but not least, the recruitment of a larger number of patients is needed for the confirmation of all findings reported in this study and to establish, if possible, a correlation between the genomic profile of BCC samples and their clinical characteristics including their subtypes and severity.

## Supplementary Information

**Additional file 1: Appendix 1**. Somatic tumor genes panel.

**Additional file 2: Appendix 2**. Identified variants and patients’ characteristics. Tier I: variants with strong clinical significance, Tier II: variants with potential clinical significance, I: Infiltrative, U: Undetermined, N: Nodular, S: Superficial, Pi: Pigmented, M: Man, W: Woman, -: Not found in gnomAD, numbers in gnomAD represent to frequencies of each variant if found in this database.

## Data Availability

All data generated or analyzed during this study are included in Additional file 2: Appendix 2 of this manuscript. The raw sequence datasets generated during the current study are not publicly available because it is possible that individual privacy could be compromised. For any further permissions to obtain access to the raw data, please contact Hampig Raphael Kourie by mail on hampig.kourie@usj.edu.lb.

## Abbreviations

AMP: Association for Molecular Pathology
ASCO: American Society of Clinical Oncology
BCC: Basal cell carcinoma
BWA: Burrows-Wheeler Aligner
CAP: College of American Pathologists
GATK: Genome Analysis Tool Kit
HDF: Hôtel Dieu de France
Hh: Hedgehog
LOH: Loss of heterozygosity
MSC: Melanoma skin cancer
NGS: Next generation sequencing
NMSC: Non melanoma skin cancer
WES: Whole exome sequencing
