# Supplementary material for: Molecular profiling of basal cell carcinomas in young patients
Source: BMC Med Genomics. 2021 Jul 20;14:187. doi: 10.1186/s12920-021-01030-w (PMC8293576; doi:10.1186/s12920-021-01030-w)
Supplement: Supplementary file 1 — Additional file 1: Appendix 1. Somatic tumor genes panel. [file 12920_2021_1030_MOESM1_ESM.docx]

**Appendix 1**

Somatic tumor genes panel:

*ABL1, AKT1, AKT2, AKT3, ALK, APC, AR, ARAF, ARID1A, ASXL1, ATM, ATR, ATRX, AXL, BAP1, BRAF, BRCA1, BRCA2, BTK, CBL, CCND1, CDH1, CDK12, CDK4, CDK6, CDKN1B, CDKN2A, CDKN2B, CHEK1, CHEK2, CREBBP, CSF1R, CTNNB1, DDR2, EGFR, ERBB2, ERBB3, ERBB4, ERCC2, ESR1, EZH2, FANCA, FANCD2, FANCI, FBXW7, FGFR1, FGFR2, FGFR3, FGFR4, FLT3, FOXL2, GATA2, GNA11, GNAQ, GNAS, H3F3A, HIST1H3B, HNF1A, HRAS, IDH1, IDH2, JAK1, JAK2, JAK3, KDR, KEAP1, KIT, KMT2A, KMT2C, KMT2D, KNSTRN, KRAS, MAGOH, MAP2K1, MAP2K2, MAP2K4, MAPK1, MAX, MDM4, MED12, MEN1, MET, MLH1, MPL, MRE11, MSH2, MSH6, MTOR, MYC, MYCN, MYD88, NBN, NF1, NF2, NFE2L2, NOTCH1, NOTCH2, NOTCH3, NRAS, NTRK1, NTRK2, NTRK3, PALB2, PDGFRA, PDGFRB, PIK3CA, PIK3CB, PIK3R1, PMS2, POLE, PPP2R1A, PTCH1, PTEN, PTPN11, RAC1, RAD50, RAD51, RAD51B, RAD51C, RAD51D, RAF1, RB1, RBM10, RET, RHEB, RHOA, RIT1, RNF43, ROS1, SETD2, SF3B1, SLX4, SMAD4, SMARCA4, SMARCB1, SMO, SPOP, SRC, STAT3, STK11, TERT, TOP1, TP53, TSC1, TSC2, TSHR, U2AF1, VHL* and *XPO1.*
